# Microbial synergy between *Rhodospirillum rubrum* and *Acetobacterium woodii* enables anaerobic CO conversion to polyhydroxyalkanoates†

**DOI:** 10.1039/d5gc01092f

**Published:** 2025-05-28

**Authors:** Timon M. Torres Ruano, Martijn Diender, Diana Z. Sousa

**Affiliations:** a Laboratory of Microbiology, Wageningen University & Research The Netherlands diana.sousa@wur.nl; b Centre for Living Technologies, EWUU Alliance The Netherlands

## Abstract

The high cost of traditional substrates has hindered the large-scale adoption of polyhydroxyalkanoates (PHAs) as sustainable alternatives to petrochemical plastics. One-carbon (C1) substrates like carbon monoxide (CO) offer a low-cost, sustainable feedstock, but efficient biocatalytic systems for their conversion to PHAs have been lacking. Here, we report the first successful anaerobic production of PHAs from CO using a synthetic co-culture of *Rhodospirillum rubrum* and *Acetobacterium woodii*. In this system, *R. rubrum* catalyzes the water–gas shift reaction, converting CO into H_2_ and CO_2_. *A. woodii* subsequently transforms these products into acetate, serving as an organic carbon source for PHA accumulation by *R. rubrum*. Neither organism, in monoculture, was able to grow on CO alone, underscoring the importance of the microbial synergy. While continuous cultivation in chemostats proved unstable, fed-batch cultivation achieved a PHA production rate of 58 ± 11 mg L_medium_^−1^ day^−1^ with a final PHA content of 38 ± 5% (dry weight). This study introduces a pioneering anaerobic route for PHA synthesis from CO, representing a significant advance toward sustainable PHA production from C1 substrates.

## Abstract 2

A novel biocatalyst for the production of polyhydroxybutyrate using carbon monoxide as the sole substrate.

## Graphical abstract

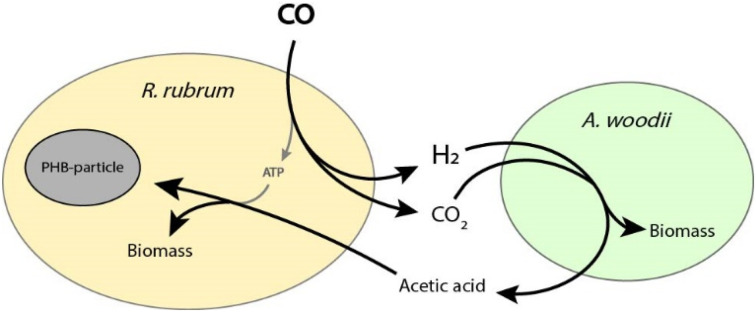

**Green foundation**

1. This work presents the proof of principle of a novel biocatalytic anaerobic process that converts CO into the biodegradable plastic precursor polyhydroxybutyrate (PHB). The innovation lies in the use of a synthetic, defined co-culture composed of a purple nonsulfur bacterium and an acetogen.

2. The co-culture achieves PHB accumulation of 38 ± 5% of cell dry weight, representing the highest PHB yield reported for anaerobic CO conversion. This achievement lays the foundation for developing a biotechnological process that converts industrial off-gases (*e.g.* steel mill off-gas) and CO-containing syngas from sustainable sources (*e.g.* biomass and waste) into biodegradable plastic precursors.

3. Future research should be directed to optimizing co-culture compositions and cultivation conditions to maximize PHB production from gaseous substrates. Testing CO-containing syngas from sustainable sources could improve both efficiency and sustainability, creating a solid basis for successful scale-up.

## Introduction

In modern society, plastics are widely utilized for numerous purposes, including packaging, coatings, and construction. The majority of plastics currently in use are derived from non-renewable petrochemical sources and exhibit poor biodegradability. Over time, various biologically-sourced plastics have been explored, such as bio-polyethylene (PE), polylactic acid (PLA), and polyhydroxyalkanoates (PHAs). These bioplastics demonstrate a range of biodegradability, from non-biodegradable to fully biodegradable.^1^ Since the 1960s, PHAs have been recognized as a promising bio-based alternative to petrochemical polyethylene. However, the high cost associated with the raw materials needed for its production has posed a significant obstacle to large-scale manufacturing.^2^

Recently, the interest in PHAs has been reignited due to their superior biodegradability compared to most other bio-based plastics.^3^ This renewed attention has spurred the search for more cost-effective raw materials for their production. C1 gases, including CH_4_, CO_2_, and CO, have gained special interest in meeting this need due to their industrial availability and low cost.^4^ In particular, CH_4_ has already been successfully used for commercial-scale production of PHAs using aerobic methanotrophs (Mango Materials, USA). The conversion of CO_2_ into PHA has been proven to be more challenging, necessitating an external energy source like hydrogen^5^ or light.^6^

Carbon monoxide shows promise as substrate for PHA production due to its high energy content and ability to support microbial metabolism through processes such as the water–gas shift reaction.^7^ A common source of CO is flue gas from the steel mill industry,^8^ but CO-containing syngas can also be generated through the gasification of renewable or waste-derived feedstocks.^9^ Currently, the sole scaled-up industrial biotechnological application of CO fermentation is for ethanol production (LanzaTech, USA). Yet, the range of products from gas fermentation is expanding, encompassing medium chain length fatty acids and higher alcohols,^10^ acetone and isopropanol,^11^ with proof-of-concept now demonstrated.

Production of PHA from CO has proven to be challenging, and is mainly hindered by the lack of native carboxydotrophic organisms capable of producing PHAs. Most PHA producing organisms are (facultative) aerobes, which can be engineered to utilize CO.^5^ These organisms however face the inherent downside that a fraction of the substrate is lost to respiration, and thus not directly used for product formation. In addition, the mixing of CO or syngas with oxygen is challenging and hazardous, making process design complex.^12^ More recently, the PHA production pathway has been introduced into natural anaerobic CO/syngas consuming acetogens.^13–15^ However, the yield optimization of these strains has proven to be challenging, with a maximum reported carbon yield of 0.47%.^14^ The third strategy attempted for the conversion of CO into PHA is through a two-stage sequential setup. In this process, CO or syngas is primarily converted into fatty acids and alcohols, and are subsequently fed to an aerobic PHA production process.^16^

Even though this process relies on two well-defined technologies, the infrastructure required to accommodate both stages complicates the process. Furthermore, PHA is produced aerobically, and therefore the loss of substrate to respiration still applies.

In order to enable production of PHAs from CO, we established a synthetic co-culture of the PHA producer *Rhodospirillum rubrum* together with the acetogen *Acetobacterium woodii*. *R. rubrum* is a well-studied purple non-sulfur bacterium, primarily known for its photoheterotrophic growth on numerous organic carbon sources.^17^ In addition, *R. rubrum* is also capable of carboxydotrophic growth using the water gas shift reaction (WGS) (Table 1, reaction (1)).^18,19^ During this process electrons from CO are directly diverted to H_2_ production and are not expected to contribute to assimilation metabolism.^20^ This explains why *R. rubrum* cannot grow autotrophically with CO as its sole substrate; in the dark, CO works as energy source and an additional organic carbon source is essential for supporting its growth. The addition of 10 mM acetate (with 1 g L^−1^ yeast extract) during growth on CO was found to stimulate growth rate and PHA accumulation of *R. rubrum* by ∼5-fold compared to yeast extract.^20^

**Table 1:** Overview of overall reactions catalysed by either *R. rubrum* (reaction (1) and (3)) or *A. woodii* (reaction (2)), with the corresponding Gibbs free energy change under standard conditions (at pH 7)

Reaction	Δ*G*°′ (kJ per reaction)
(1) CO + H_2_O → H_2_ + HCO_3_^−^ + H^+^	−15.2
(2) 4H_2_ + 2HCO_3_^−^ + H^+^ → acetate + 4H_2_O	−104.6
(3) 9 acetate + 7 H^+^ → 4 3-hydroxyburyate + 2HCO_3_^−^	+ 404.9
(4) 9CO + 9H_2_O → 3-hydroxybutyrate + 5HCO_3_^−^ + 5H^+^	−270.9

**Fig. 1:**
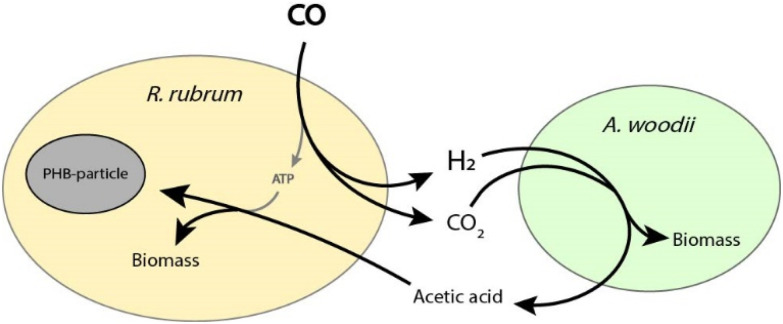
Schematic overview of a synthetic co-culture of *R. rubrum* and *A. woodii*. Reactions are not displayed stoichiometrically.

By co-cultivation of *R. rubrum* with the acetate-producing *A. woodii*, we aimed to lift the requirement of an external organic carbon source for *R. rubrum*. The complementary nature of the water–gas-shift reaction of *R. rubrum* (Table 1, reaction (1)) with the H_2_ + CO_2_ driven acetogenic Wood-Ljungdahl pathway of *A. woodii* (Table 1, reaction (2)), theoretically solves the organic carbon requirement *R. rubrum* for growth and PHA synthesis (Table 1, reaction (3) and Fig. 1), with a maximum theoretical yield according to reaction (4) (Table 1). Synthetic co-culture approaches have previously been shown to expand the product spectrum of syngas fermentation, enabling production of *e.g.* medium chain fatty acids, medium chain alcohols, and biomethane.^21,22^ Furthermore, a co-culture of a purple non-sulfur bacterium with an acetogen has previously been reported for the cross-feeding of ammonia.^23^ Here, we provide proof-of-concept for the co-culture of *R. rubrum* and *A. woodii*, enabling the anaerobic production of PHAs, specifically poly-3-hydroxybutyrate (PHB), from CO as sole substrate. A PHA production rate of 58 ± 11 mg L^−1^ day^−1^ was achieved by this co-culture, with a final PHA content of 38 ± 5% of the dry weight.

## Experimental

### Microorganisms and culture conditions

*Rhodospirillum rubrum S1* (DSM 467) and *Acetobacerium woodii* (DSM 1030) were purchased from DSMZ (Braunschweig, Germany). Cultivation medium contained per litre: 0.9 g NH_4_CL, 0.2 g MgSO_4_·7H_2_O, 0.050 g KH_2_PO_4_, 0.02 g CaCl_2_, 0.1 g yeast extract and 0.5 mg resazurin. The medium was supplemented with trace elements (per litre of medium): 1.5 mg FeCl_2_·4H_2_O, 0.025 mg FeCl_3_·6H_2_O, 0.070 mg ZnCl_2_, 0.10 mg MnCl.4H_2_O, 0.006 mg H_3_BO_3_, 0.190 mg CoCl_2_·6H_2_O, 0.002 mg CuCl_2_·2H_2_O, 2.6 mg NiCl_2_·6H_2_O and 1.0 mg Na_2_MoO_4_·2H_2_O, 0.0035 mg Na_2_SeO_3_, and 0.2 mg Na_2_WO_4_. 45 mL of medium was dispensed into 550 mL serum Bottles, closed with butyl rubber caps and sealed with aluminium crimp caps. The headspace was then flushed and replaced with 600 mbar CO and 400 mbar CO_2_, after which the bottles were autoclaved (120 °C, 20 min). After autoclavation, the medium was reduced by adding 2%v/v of a solution containing 1 M KHCO_3_ + 20 mM Na_2_S.9H_2_O solution. Additionally, medium was supplemented with 1% v/v of vitamin solution containing (per litter): 1 mg biotin, 10 mg nicotinamide, 5 mg *p*-aminobenzoic acid, 10 mg riboflavin, 5 mg pantothenic acid, 25 mg pyridoxamine and 5 mg cyanocobalamine. The final pH of the medium was between 6.7 and 6.9. For cultivation of pure cultures of *A. woodii* medium was amended with 50 mM NaCl and CO was substituted with H_2_.

### Bioreactor experiments

Two reactor systems were used in this work. Initial chemostat experiments were conducted using two 700 mL vessels (HEL Group, Hemel Hempstead, UK) with a working volume of 550 mL. For the fed-batch experiments, we utilized a Mini-Bio® bioreactor system (Applikon, Delft, The Netherlands), comprising 3 vessels each with a capacity of 1200 mL and filled with 1100 mL medium. Both reactor systems were equipped with probes for pH and oxidation reduction potential. The pH of the cultivation medium was controlled and maintained at 7.0 ± 0.1 (by adding a solution of 3 M KOH or 2 M HCl). Continuous gas inflow was maintained by employing 3 mass flow controllers per reactor vessel, supplying a gas mixture consisting of 60% CO, 25% N_2_ and 15% CO_2_ by volume, at a total flow rate 2% v/v per min. The stirring speed increased incrementally over time: from 100 rpm (*t* = 0–1 day) to 200 rpm (*t* = 2–3 days), then to 450 rpm (*t* = 4–5 days), and ultimately to 550 rpm for the remainder of the experiment (*t* > 5). The medium used in the bioreactor experiments was identical to the one used for batch bottle cultivation, with the exception of Na_2_S·9H_2_O which was substituted by 0.1 g L^−1^l-cystein-HCl.

### Analytical techniques

Organic acids were measured by high pressure liquid chromatography (Prominence-I LC 2030C, Agilent Technologies, Santa Clara, CA). A HIPLEX H column (300 × 7.7 mm Agilent Technologies, Santa Clara, CA) at 45 °C and an eluent flow rate of 1.0 mL min^−1^ 0.005 M H_2_SO_4_ equipped with an RI detector (RID-20A, Shimadzu, Kyoto, Japan). Samples were centrifuged for 2 min at 13 000*g* and acidified with 1 : 1 ratio of 0.05 M H_2_SO_4_ before injection.

The sodium ion concentration was measured using ion chromatography on a Thermo ICS2100 (Thermo Fischer Scientific, Waltham, MA) equipped with a Dionex Ionpac CS18 column, 250 mm x 2 mm, set to 30 °C. The eluent consisted of 4 mM methanesulfonic acid. The flow was set at 0.30 ml min^−1^. Detection was done with a non-suppressed conductivity detector.

A Compact GC 4.0 (Global Analyser Solution, Breda, The Netherlands) was used to measure H_2_, CO and CO_2_ partial pressure in the headspace of both the bottles and reactors. In batch bottles 0.2 mL samples were diluted to 1 mL with air and allowed to equilibrate for 1–10 min before injection. Gas samples from reactor vessel were not diluted before injection. During injection the samples were loaded onto two channels, both containing their own sample loop (25 μL each). Channel one detected H_2_ and CO by using a Carboxen 1010 3 m × 0.32 mm pre column for water separation followed by a Molsieve 5A column. Both columns operated at 100 °C with argon flow at 1 mL min^−1^. Channel two detected CO_2_, and contained a Rt-Q-BOND 10 m × 0.32 mm column operated at 80 °C with 1 mL min^−1^ argon flow. Both channels contained their own thermal conductivity detector.

Biomass was quantified by measuring the optical density of cultures at 600 nm with a Shimadzu UV-1800 spectrophotometer. For dry weight determination, 4–14 mL culture was centrifuged in a 15 mL tube at 3780 *g* for 15 min. The supernatant was discarded and the pellet was washed by resuspension in 5 mL demi water (2×). The pellet was resuspended in 1 mL demi water and transferred to a pre-weight aluminium drying cup. The cup contained ¼ of a 55 mm glass Whatman microfibre filter GF/F (Cytiva, Marlborough, MA). The piece filter was added to prevent biomass from flacking during the overnight drying. The dried cups were weight again to determine weight gain.

The PHA concentration and composition was monitored over time by collecting the pellet of 1–2 mL samples (after centrifugation at 13 000*g* for 3 min). Approximately 1 mL of the supernatant was collected for liquid analysis. The remaining supernatant was discarded and the pellet stored at −20 °C until further analysis. Upon measuring, the samples were thawed, resuspended in 1 mL methanol : HCl (37%) 4 : 1 and sonicated for 3 min. Benzoic acid (2 g L^−1^) was used as internal standard, with 20 μL added to the sample. Samples were vortexed and transferred to 25 mL glass vials where 1 mL chloroform was added. The vials were vortexed and incubated at 100 °C for 4 hours. After 2 h the samples were vortexed again. After samples cooled to room temperature, 0.5 mL of demi water was added to each sample and vortexed three times at 2 min intervals. Samples were centrifuged at 100*g* for 3 min to separate the solvent and water phase. From the solvent phase 100 μL was transferred to a 1.5 mL HPLC vial with glass insert and a PTFE lined screw cap. From these vials 0.5 μL was injected on a gas chromatograph (Thermo Scientific Trace 1300) equipped with a DB-WX UI column (30 m × 0.25 mm, Agilent Technologies). The injection temperature was set to 250 °C and the oven temperature was ramped from 50–200 °C at 15 °C min^−1^. H_2_ was used as carrier gas at a flow rate 1 mL min^−1^. Detection was performed with a mass spectrometer (Thermo Scientific ISQ700). Values reported here for PHB are in mg per liter culture volume.

## Results & discussion

**Fig. 2:**
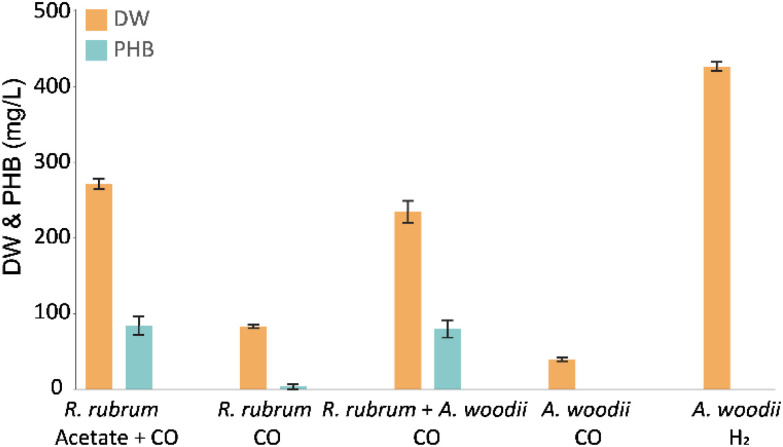
Cell dry weight and PHA accumulation (in mg L_medium_^−1^) after 5 days of cultivation. *R. rubrum* monoculture with and without 18 mM potassium acetate and CO as sole energy source (*n* = 5); co-culture of *R. rubrum* and *A. woodii* with CO as sole energy and carbon source (*n* = 5); and, *A. woodii* in mono-culture with H_2_ or CO (*n* = 4). Error bars indicate standard deviation. Note that to all conditions 0.4 bar of CO_2_ was provided as (secondary) carbon source.

To evaluate carboxydotrophic PHB production by the co-culture of *R. rubrum* and *A. woodii*, anaerobic batch bottle cultivation was performed (Fig. 2). In order to rule out PHB production by *R. rubrum* on solely CO, growth and PHB production were tracked in monocultures with and without supplemented acetate. The results confirm the drastic increase in growth and PHB accumulation when acetate is provided as organic carbon source. When acetate was substituted for *A. woodii*, this did not significantly affect the PHB accumulation (*p* = 0.61), showing the acetogen performs its expected role.

Due to carryover of acetate from the *A. woodii* inoculum, 1.2 ± 0.3 mM acetate was present at the start of the co-cultivation. However, during mono-culture cultivation of *R. rubrum* 5.0 ± 0.3 acetate was consumed. Thus, acetate had to be produced *in situ* to sustain a similar level of PHB accumulation in the co-culture. Activity of *A. woodii* in the co-culture was additionally reflected in the net H_2_ production. From the initially supplied 14.4 mmol of CO, the co-culture produced 10.0 ± 0.5 mmol H_2_ compared to the 12.2 ± 0.3 mmol produced by *R. rubrum* in mono-culture with acetate. These results also confirm that H_2_ is the preferred substrate for *A. woodii* compared to CO, as was previously reported.^24^ A low CO affinity of the acetogen is beneficial for this specific co-culture, as it helps prevent substrate competition. Additionally, the strong carboxydotrophic capacity of *R. rubrum* lowers available CO in the liquid, minimizing potential inhibitory effect on the metabolism of *A. woodii*.

### Chemostat cultivation results in an unstable co-culture and varying PHB production

To increase PHB production, and assess the stability of the co-culture, chemostat cultivation was performed. Two chemostat setups were supplied with CO and CO_2_ as energy and carbon source, without acetate in the inflow. Both systems were initiated by establishing a *R. rubrum* mono-culture steady state, resulting in an average biomass concentration of 190 ± 20 mg L^−1^ over three dilutions.

**Fig. 3:**
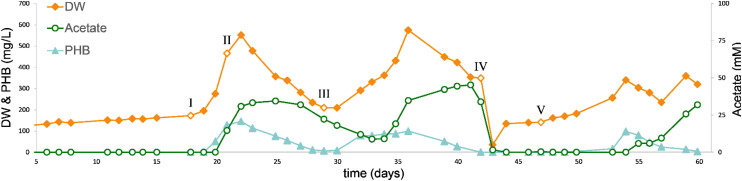
Biomass and product concentration of a continuous culture of *R. rubrum* in mono-culture (*t* < 18 days) and in co-culture with *A. woodii* (*t* > 18 days) with a dilution rate of 0.25 day^−1^. I: Addition of *A. woodii*, II: decreasing sodium concentration of inflow from 5 to 2.5 mM, III: re-inoculation of *R. rubrum*, IV: decreasing sodium concentration of inflow from 2.5 to 0 mM, rinsing with 5 reactor volumes and re-inoculation of *R. rubrum*, V: re-inoculation of *A. woodii*.

During this initial steady state, only H_2_, CO_2_ and biomass were produced, and no PHB formation could be detected (Fig. 3). It cannot be concluded that this biomass was autotrophically synthesized from CO_2_/CO, as the biomass concentration did not exceed the organic carbon supplied in the form of yeast extract and cysteine–HCl (both 0.1 g L^−1^). On day 18 of chemostat cultivation, 10 mL of active *A. woodii* culture was inoculated into one of the systems (Fig. 3, point I). The second reactor was maintained with *R. rubrum* as control, operating as a mono-culture in steady state for the remainder of the experiment (Fig. S1†). The addition of *A. woodii* resulted in an increase in the biomass, PHB and acetate concentrations to 520 mg L^−1^, 150 mg L^−1^ and 40 mM, respectively. The accumulation of both PHB and acetate shows that both strains are simultaneously active in the co-culture. The observed accumulation of acetate is, however, expected to negatively affect the growth of *R. rubrum* above 18 mM.^25^ It was subsequently aimed to reach a more favourable balance in the culture by lowering the metabolic rate of *A. woodii*.

Acetogenic activity of *A. woodii* is known to be sodium dependent, due to its sodium ion translocating RnF complex and ATP-synthase.^26–28^ Sodium limitation can be applied to reduce the rate of acetogenic activity in *A. woodii*.^29^ Therefore, the sodium ion concentration in the inflow of both chemostats was decreased from 5.4 ± 0.2 mM to 2.4 ± 0.2 mM (t = 21 days) (Fig. 3, point II). The *R. rubrum* mono-culture was not affected by this change, yet the co-culture responded with a rapid decrease in biomass and PHB concentration. After 8 days of biomass and PHB decline, the system was re-inoculated with 20 mL of active *R. rubrum* culture (Fig. 3, point III). This resulted in a second biomass and PHB accumulation phase, peaking at 580 mg L^−1^ biomass and 100 mg L^−1^ PHB on day 36. During part II and III, despite the lowered sodium ion concentration in the inflow, acetate still accumulated up to 45 mM. Biomass and PHB concentrations decreased after peaking on day 36, again without achieving steady state.

In a third attempt to stabilize the co-culture in chemostat setting, the liquid inflow was replaced with a ‘sodium ion free’ medium. It should be noted that this medium still contained 100 mg L^−1^ yeast extract, resulting in 0.7 ± 0.0 mM of sodium ions still being present. The co-culture reactor was rinsed with 5 volumes of the new medium to remove the remaining sodium ions. Following the rinsing step, the reactor was re-inoculated with 30 mL of active, sodium ion free, *R. rubrum* culture. *A. woodii* was inoculated 4 days later (10 mL, 5 mM sodium ions in inoculum). Similar to the previous two attempts, there was biomass and PHB accumulation, peaking at 340 mg L^−1^ biomass and 100 mg L^−1^ PHB, followed by wash-out of biomass and PHB (Fig. 3, point V). A steady state with this co-culture could thus not be achieved despite the attempt to control the activity of *A. woodii*. As anaerobic PHB consumption by *R. rubrum* during growth on CO and acetate has not been reported, wash out of *R. rubrum* is the most likely cause for the observed decrease in PHB. The wash out indicates a sudden decrease in the growth rate of *R. rubrum*. A similar physiological switch was observed by Karmann and colleagues during a fed-batch cultivation of *R. rubrum*, grown on CO and acetate. In their work cell division stopped upon the start of PHB accumulation.^30^ In a chemostat this would result in wash out, explaining the observed behaviour. Additionally, acetate toxicity could have a negative effect on the *R. rubrum* growth rate. To assess the effect of acetate inhibition, 32 batch bottles of *R. rubrum* were cultivated with up to 175 mM acetate and CO. A negative linear relation was found between initial acetate concentration and CO consumption rate. However, at 40 mM acetate the activity of *R. rubrum* only decreased by only 12% (ESI Fig. S2†). Acetate inhibition is thus not expected to be a major factor in the washout of *R. rubrum* observed in the chemostat system.

### Fed-batch co-cultivation allows for PHB production from solely CO

As washout of *R. rubrum* appeared to be a major issue during chemostat cultivation, bioreactors were operated in fed-batch mode to assess the PHB production potential of the co-culture approach. Reactors were inoculated with *R. rubrum* and 10 mM of potassium acetate was added as the organic carbon source to promote initial growth. Upon depletion of all initial acetate (*t* = 5 days), 10 mL active *A. woodii* culture (OD_600_: 0.305, 33 mM acetate) was inoculated into the system. With the inoculum of *A. woodii*, sodium ions were carried over, resulting in a final sodium ion concentrations of 3.9–5.1 mM. As a control condition reactors were operated with mono-cultures of *R. rubrum* in identical operation conditions.

**Fig. 4:**
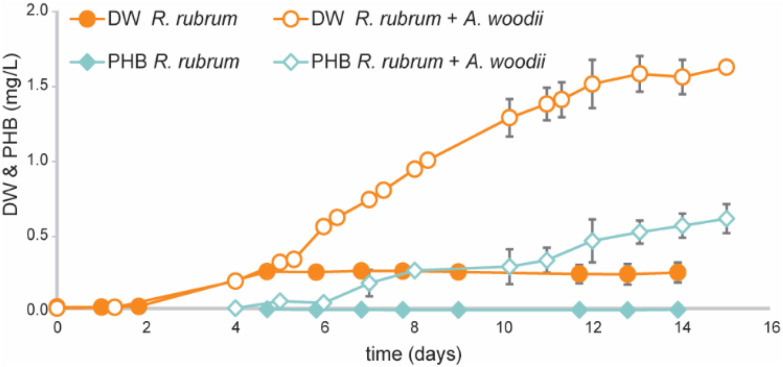
Biomass and PHA concentration in fed-batch cultivations of mono-culture *R. rubrum* (*n* = 2) compared to a co-culture of *R. rubrum* and *A. woodii* (*n* = 3).

**Fig. 5:**
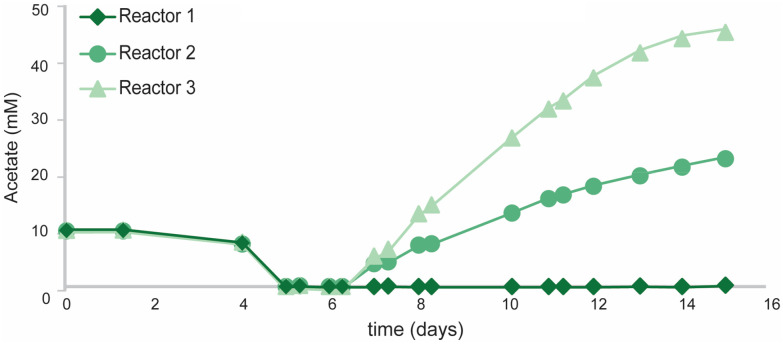
Acetate accumulation in fed batch cultivations of the three replicate co-cultures of *R. rubrum* and *A. woodii*.

During the first phase of growth (*t* = 0–5 days) all reactors showed similar growth profiles (Fig. 4) and only trace amounts of PHB were measured (<10 mg L^−1^). After 5 days, all initial acetate was consumed and subsequently growth halted in the mono-culture reactors. Water–gas-shift activity was still observed until the end of the operation (ESI Fig. S3†), yet no PHB accumulation was observed during this phase of cultivation. Even though *R. rubrum* has been proposed to grow with CO as sole energy (*i.e.* in the dark) and carbon source,^31^ it was never demonstrated that the obtained biomass concentration exceeded that of supplied yeast extract. Strictly autotrophic growth was therefore never confirmed. The inability of *R. rubrum* to grow autotrophically on CO was already predicted when its transcriptome was analysed during growth on CO and acetate.^20^ The arrest in growth upon organic carbon depletion, as observed here, strongly supports this. In contrast to the mono-culture, the co-culture showed steady growth during fed-batch cultivation, even though no acetate could be detected for the first 31 hours (Fig. 5). The continuation of growth in the co-culture suggest the *in situ* production of organic carbon by *A. woodii*. Both biomass and PHB concentrations increased at average rate of 130 ± 20 and 58 ± 11 mg L_medium_^−1^ day^−1^, respectively, during the 10 day co-culture phase. Final biomass and PHB concentrations were 1620 ± 30 and 610 ± 10 mg L^−1^, respectively. The total PHA fraction reached up to 38 ± 5% of cell dry weight and contained >98% PHB. Though consistent growth and PHB accumulation was obtained between the three co-culture replicates, a large variation in acetate accumulation was observed (Fig. 5). The fact that no variation in growth or PHB accumulation was observed between the triplicates contradicts the findings by Najafpour and Younesi,^25^ who observed a decrease growth rate in a phototrophic *R. rubrum* culture above 18 mM acetate.

Electron balances of the three fed-batch reactors show that 1.5 to 2.3% of electrons derived from CO were directed to PHB production. Hydrogen was a major electron sink, taking up 86 to 91% of available electrons (Table 2). Carbon balancing shows that PHB only comprises 0.7–1.0% of the total supplied carbon from CO, whereas CO_2_ was the main carbon containing product with 84 to 91% of carbon from CO being converted to CO_2_. This shows that the current bottleneck of the co-culture is limited acetate uptake by *R. rubrum* per water–gas shift reaction.

**Table 2:** Electron and carbon balance over the average rates during the co-culture phase (*t* = 5–15 days) in the fed-batch reactors (R1–3)

Component	Electron balance			Carbon balance		
	R1 (%)	R2 (%)	R3 (%)	R1 (%)	R2 (%)	R3 (%)
Acetate	0.0	3.1	7.4	0.0	1.5	3.7
H_2_	90.7	91.4	85.8	—	—	—
CO_2_	—	—	—	84.3	87.7	90.9
PHB	1.5	2.3	2.2	0.7	1.0	1.0
PHB free biomass	2.1	2.2	1.8	0.9	1.0	0.8
Total completeness	94.3	99.0	97.2	85.9	91.2	96.4

Activation of acetate to the acetyl-CoA in *R. rubrum* is proposed to be coupled to the oxidative tri-carboxylic acid cycle through a succinate-transferase.^32^ The reducing equivalents and CO_2_ obtained during this oxidative pathway are re-assimilated through the Calvin-Benson cycle,^33^ making assimilation of biomass from acetate ATP intensive. Combined with the low energy potential from the biological water gas shift reaction,^7^ this results in a low acetate consumption per CO conversion. Revelles and colleagues^20^ demonstrated that the growth rate of *R. rubrum* with CO and acetate could be improved by the addition of light as secondary energy source. A similar strategy could be applied to the co-culture to improve the growth and PHB accumulation rates.

Other systems converting CO to PHB have been reported in literature, including mono-cultures of *R. rubrum* co-fed with acetate, engineered acetogens, two-stage systems involving an acetogenic step followed by a PHA accumulation step, and aerobic processes (Table 3). When comparing the synthetic co-culture approach described here to other anaerobic processes, the observed PHA accumulation – both in terms of rate and final PHA accumulation – is generally higher – particularly under conditions where CO is the sole substrate. Karmann *et al.*^30^ reported a 20% higher PHA accumulation rate in mono-cultures of *R. rubrum*, but their system relied on acetate supplementation and a CO flow rate more than twice that used in the present study. Another interesting observation in the work of Karmann *et al.*^30^ is the dramatic increase in PHA accumulation during phosphate limitation, by a factor 4.6. While this strategy could potentially be applied to the synthetic co-culture, the impact of nutrient limitation in *A. woodii* remains unknown and was not explored here. Higher PHA accumulation rates have also been reported for aerobic CO-utilizers, which oxidize CO and H_2_ as energy sources. Although a direct comparison is not possible due to the lack of reported CO and H_2_ feed flow rates in those studies, it is reasonable assume these systems may achieve higher PHA accumulation rate due to their high ATP yielding respiratory metabolism. However, the addition of O_2_ to CO/H_2_ mixtures created flammable conditions, posing significant operational and safety challenges.^12^ A major drawback of aerobic CO-utilizers is that they oxidize CO to CO_2_ to generate energy. Although CO_2_ can be fixed by these microorganisms *via* the Calvin–Benson cycle, this process requires a significant amount of ATP, meaning CO_2_ fixation comes with a high energy cost, and much of the CO_2_ may still be lost. In contrast, the co-culture system described here benefits from the energy-efficient Wood–Ljungdahl pathway used by acetogens like *A. woodii*. In this co-culture system, CO_2_ released by *R. rubrum* during CO metabolism is captured and fixed by *A. woodii*, abolishing the requirement of the Calvin-Benson cycle as CO_2_ fixation pathway. This internal recycling of CO_2_ has the potential to improve overall carbon retention in the final product, providing a clear advantage over aerobic processes.

**Table 3:** Literature overview of PHA production from CO

Organism	Operation mode	Anaerobic	CO H_2_ rate (mL L^−1^ min^−1^)	Substrate	Yeast extract	Final PHA (g L^−1^)	Final PHA content (%)	PHA accumulation rate, *r*_PHA_ (mg L^−1^ day^−1^)	Ref.
**Anaerobic processes**									
*R. rubrum* co-culture with acetogens									
*R. rubrum* + *A. woodii*	Fed-batch	y	CO: 12	CO	0.1 g L^−1^	0.61 ± 001	38 ± 5%	58 ± 11	This work
*R. rubrum* mono-culture									
*R. rubrum*	Fed-batch	y	CO: 15	CO + acetate	1 g L^−1^	0.63^a^	38%	59.2	(Do *et al.*, 2007)^19^
*R. rubrum*	Fed-batch	y	CO: 15	CO	1 g L^−1^	nr	nr	11.5	(Do *et al.*, 2007)^19^
*R. rubrum*	Fed-batch	y	CO: 25	CO + acetate	1 g L^−1^	0.59	11%	69.6	(Karmann *et al.*, 2019)^30^
*R. rubrum*	Fed-batch P-limited	y	CO: 25	CO + acetate	1 g L^−1^	1.6	30%	325	(Karmann *et al.*, 2019)^30^
*R. rubrum*	Batch	y	—	CO + acetate	None	nr	28 ± 10%	—	(Revelles *et al.*, 2016)^20^
*R. rubrum*	Batch	y	—	CO	1 g L^−1^	nr	20 ± 15%	—	(Revelles *et al.*, 2016)^20^
*R. rubrum*	Batch	y	—	CO + succinate	0.3 g L^−1^	nr	10.1 ± 1%	—	(Heinrich *et al.*, 2015)^34^
Engineered acetogens									
*C. coskatii*	Batch	y	—	CO	0,5 g L^−1^	nr	1.2 ± 0.1%	—	(Flüchter *et al.*, 2019)^13^
*C. ljungdhali*	Batch	y	—	CO	0,5 g L^−1^	nr	1.2 ± 0.1%	—	(Flüchter *et al.*, 2019)^13^
*C. autoethanogenum*	Chemostat	y	CO:29, H_2_: 12	CO + H_2_	None	0.0225	5.60%	1	(Lemgruber *et al.*, 2019)^14^
2-Stage systems									
*C. autoetanogenum* + mixed microbial community	2-Stage Fed-batch	Stage 1: y	CO:2.5, H_2_:1.7	CO + H_2_	1 g L^−1^	nr	24%	—	(Lagoa-Costa *et al.*, 2017)^16^
		Stage 2: n							
*C. carboxidivorans* + mixed microbial community	2-Stage Fed-batch	Stage 1: y	CO:2.5 H_2_:1.7	CO + H_2_	1 g L^−1^	nr	41.5%	7^b^	(Portela-Grandío *et al.*, 2021)^35^
		Stage 2: n							
**Aerobic processes**									
*C. necator*	Batch	y	nr	CO + H_2_	None	1.3	49.7%	52	(Heinrich *et al.*, 2018)^36^
*S. carboxydohydrogena* Z-1062	Fed-batch	n	nr	H_2_ + CO + CO_2_	None	nr	61.7%	4080	(Volova *et al.*, 2015)^5^
	N- and S-limited Fed-batch								
*S. carboxydohydrogena* Z-1062	Fed batch	n	nr	H_2_ + CO + CO_2_	None	nr	8.9%	720	(Volova *et al.*, 2015)^5^

a Calculated from reported data.

b Calculated over both stages combined.

The current challenge in the reported co-culture system is that the majority of electrons and carbon are diverted to water–gas shift products (Table 2), which negatively impacts sustainability parameters like E-factor (kg product per kg waste) and percentage yield (product/substrate).^37^ Theoretically, 9 moles of CO can produce 1 mole of PHB (Table 1, reaction (4)), corresponding to a maximum carbon yield of ∼44%. While this value is unlikely to be achieved in practice due to cellular requirements for other biomass components, the current co-culture achieves a carbon recovery of only ∼1% in PHB (Table 2), leaving substantial space for improvement. Strategies such as applying nutrient limitation, or increasing energy availability to *R. rubrum* (*e.g.*, through light, as shown in other studies – Table 3) could improve this. Nonetheless, despite the current limitations, the unoptimized co-culture system performs comparably to anaerobic systems co-fed with both CO and an organic substrate (Table 3), representing a promising starting point for developing a one-stage, CO-fed PHA production system.

## Conclusions

Anaerobic conversion of CO to PHB was achieved by co-cultivation of *R. rubrum* and *A. woodii* without the requirement of secondary carbon source or electron acceptor. Co-cultivation proved to be unstable during chemostat cultivation, yet was successful in batch and fed-batch conditions. The PHB accumulation rate obtained during the fed-batch cultivation (58 ± 11 mg L^−1^ day^−1^) was comparable to rates obtained by *R. rubrum* fed-batches grown with CO and acetate in literature. This is a proof-of-concept that the synthetic co-culture approach can be a suitable approach to convert waste gases into bioplastic precursors. This work demonstrates a promising new approach for the production of PHB from CO, setting the ground for process optimizations that will boost rate and yield toward industrial implementation.

## Conflicts of interest

The authors declare that the co-culture concept for PHA production is detailed in their patent application (WO 2024/236121), which also explores potential industrial applications.

## Supplementary Material

GC-027-D5GC01092F-s001

## Data Availability

All data underlying the results are available as part of the article and ESI† and no additional source data are required. Raw data will be made available on request to the authors.

## References

[cit1] Emadian S. M., Onay T. T., Demirel B. (2017). Waste Manage..

[cit2] Możejko-Ciesielska J., Kiewisz R. (2016). Microbiol. Res..

[cit3] Meereboer K. W., Misra M., Mohanty A. K. (2020). Green Chem..

[cit4] Yoon J., Oh M. K. (2022). Bioresour. Technol..

[cit5] Volova T. G., Kalacheva G. S., Altukhova O. V. (2001). Microbiology.

[cit6] Yashavanth P. R., Das M., Maiti S. K. (2021). J. Environ. Chem. Eng..

[cit7] Diender M., Stams A. J. M., Sousa D. Z. (2015). Front. Microbiol..

[cit8] Razzaq R., Li C., Zhang S. (2013). Fuel.

[cit9] Shahabuddin M., Alam M. T., Krishna B. B., Bhaskar T., Perkins G. (2020). Bioresour. Technol..

[cit10] Sun X., Atiyeh H. K., Huhnke R. L., Tanner R. S. (2019). Bioresour. Technol. Rep..

[cit11] Liew F. E., Nogle R., Abdalla T., Rasor B. J., Canter C., Jensen R. O., Wang L., Strutz J., Chirania P., De Tissera S., Mueller A. P., Ruan Z., Gao A., Tran L., Engle N. L., Bromley J. C., Daniell J., Conrado R., Tschaplinski T. J., Giannone R. J., Hettich R. L., Karim A. S., Simpson S. D., Brown S. D., Leang C., Jewett M. C., Köpke M. (2022). Nat. Biotechnol..

[cit12] Karmann S., Follonier S., Egger D., Hebel D., Panke S., Zinn M. (2017). Microb. Biotechnol..

[cit13] Flüchter S., Follonier S., Schiel-Bengelsdorf B., Bengelsdorf F. R., Zinn M., Dürre P. (2019). Biomacromolecules.

[cit14] de Souza Pinto Lemgruber R., Valgepea K., Tappel R., Behrendorff J. B., Palfreyman R. W., Plan M., Hodson M. P., Simpson S. D., Nielsen L. K., Köpke M., Marcellin E. (2019). Metab. Eng..

[cit15] Höfele F., Dürre P. (2023). Fermentation.

[cit16] Lagoa-Costa B., Abubackar H. N., Fernández-Romasanta M., Kennes C., Veiga M. C. (2017). Bioresour. Technol..

[cit17] Alloul A., Blansaer N., Cabecas Segura P., Wattiez R., Vlaeminck S. E., Leroy B. (2023). Trends Biotechnol..

[cit18] Kerby R. L., Ludden P. W., Roberts G. P. (1995). J. Bacteriol..

[cit19] Do Y. S., Smeenk J., Broer K. M., Kisting C. J., Brown R., Heindel T. J., Bobik T. A., DiSpirito A. A. (2007). Biotechnol. Bioeng..

[cit20] Revelles O., Tarazona N., García J. L., Prieto M. A. (2016). Environ. Microbiol..

[cit21] Diender M., Parera Olm I., Sousa D. Z. (2021). Curr. Opin. Biotechnol.

[cit22] Parera Olm I., Sousa D. Z. (2022). Adv. Biochem. Eng. Biotechnol..

[cit23] Cestellos-Blanc S., Chan R. R., Shen Y. X., Kim J. M., Tacken T. A., Ledbetter R., Yu S., Seefeldt L. C., Yang P. (2022). Proc. Natl. Acad. Sci. U. S. A..

[cit24] Bertsch J., Müller V. (2015). Appl. Environ. Microbiol..

[cit25] Najafpour G. D., Younesi H. (2007). World J. Microbiol. Biotechnol..

[cit26] Heise R., Muller V., Gottschalk G. (1992). Eur. J. Biochem..

[cit27] Kumar A., Roth J., Kim H., Saura P., Bohn S., Reif-Trauttmansdorff T., Schubert A., Kaila V. R. I., Schuller J. M., Müller V. (2025). Nat. Commun..

[cit28] Schuchmann K., Müller V. (2014). Nat. Rev. Microbiol..

[cit29] Heise R., Muller V., Gottschalk G. (1989). J. Bacteriol..

[cit30] Karmann S., Panke S., Zinn M. (2019). Front. Bioeng. Biotechnol..

[cit31] Drennan C. L., Heo J., Sintchak M. D., Schreiter E., Ludden P. W. (2001). Proc. Natl. Acad. Sci. U. S. A..

[cit32] Leroy B., De Meur Q., Moulin C., Wegria G., Wattiez R. (2015). Microbiology.

[cit33] McCully A. L., Onyeziri M. C., Lasarre B., Gliessman J. R., McKinlay J. B. (2020). Microbiology.

[cit34] Heinrich D., Raberg M., Steinbüchel A., Steinbüchel S. (2015). FEMS Microbiol. Lett..

[cit35] Portela-Grandío A., Lagoa-Costa B., Kennes C., Veiga M. C. (2021). J. Environ. Chem. Eng..

[cit36] Heinrich D., Raberg M., Steinbüchel A. (2018). Microb. Biotechnol..

[cit37] Anastas P. T. and Warner J. C., Green Chemistry: Theory and Practice, Oxford University Press, Oxford, 2000

