# Supplementary material for: Microbial synergy between Rhodospirillum rubrum and Acetobacterium woodii enables anaerobic CO conversion to polyhydroxyalkanoates
Source: Green Chem. 2025 May 28;27(26):7878–86. doi: 10.1039/d5gc01092f (PMC12152849; doi:10.1039/d5gc01092f)
Supplement: GC-027-D5GC01092F-s001 [file GC-027-D5GC01092F-s001.pdf]

Supplementary Information (SI) for Green Chemistry.
This journal is © The Royal Society of Chemistry 2025

Supplementary figures

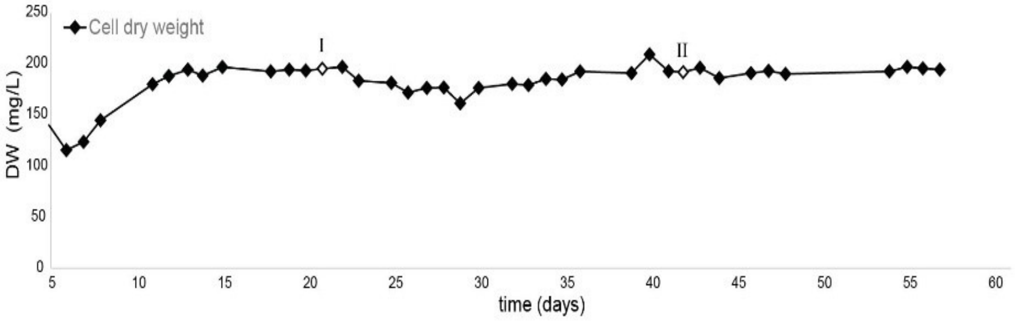

Figure S1. Biomass concentration during chemostat cultivation of a mono-culture of *R. rubrum*. I: Decreasing the sodium ion concentration of inflow from 5 to 2.5 mM, II Decreasing the sodium ion concentration of the inflow from 2.5 to 0 mM.

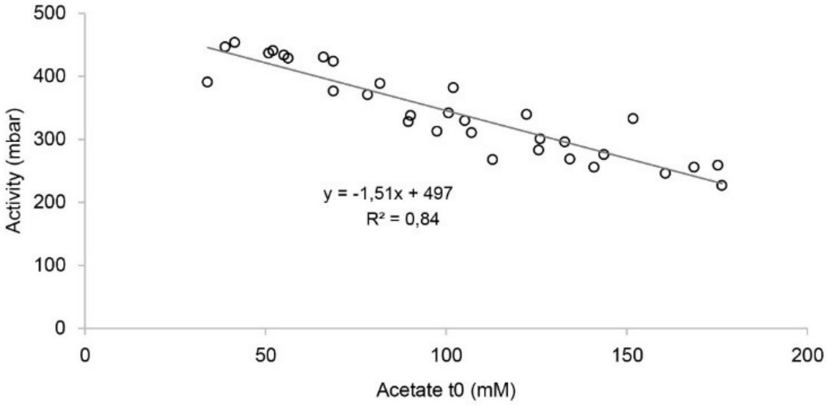

Figure S2. Activity of *R. rubrum* under a gradient of potassium acetate concentrations. Activity was measured by the pressure increase due to WGS activity at day 6 after inoculation.

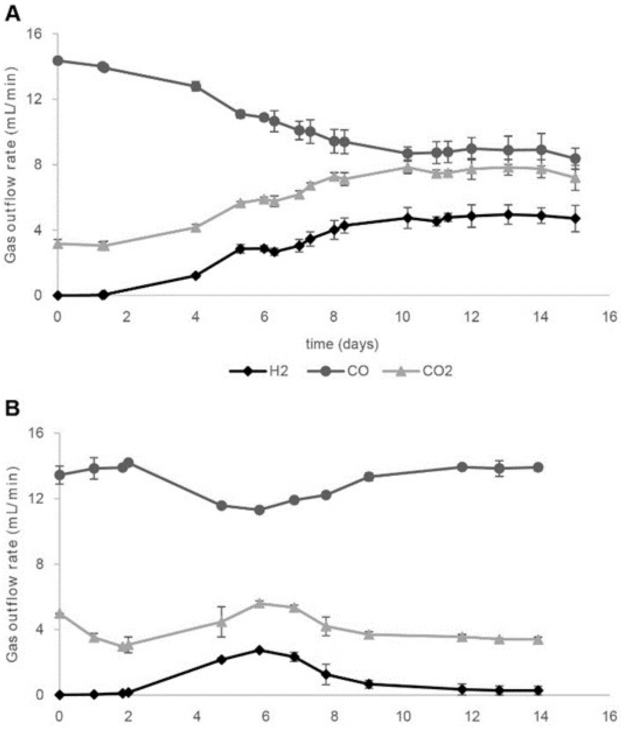

Figure S3. Gas outflow composition of fed-batch bioreactors operated with a co-culture of *R. rubrum* and *A. woodii* (A) or a mono-culture of *R. rubrum* (B).
